# Changes in employment status over time in multiple sclerosis following a first episode of central nervous system demyelination, a Markov multistate model study

**DOI:** 10.1111/ene.16016

**Published:** 2023-08-13

**Authors:** Amin Zarghami, Valery Fuh‐Ngwa, Suzi B. Claflin, Ingrid van der Mei, Anne‐Louise Ponsonby, Simon Broadley, Steve Simpson‐Yap, Ausimmune/AusLong Investigator Group, Robyn Lucas, Keith Dear, Anne‐Louise Ponsonby, Ingrid van der Mei, Leigh Blizzard, Steve Simpson‐Yap, Bruce V. Taylor, Simon Broadley, Trevor Kilpatrick, David Williams, Jeannette Lechner‐Scott, Cameron Shaw, Caron Chapman, Alan Coulthard, Patricia Valery, Bruce V. Taylor

**Affiliations:** ^1^ Menzies Institute for Medical Research University of Tasmania Hobart Tasmania Australia; ^2^ The Florey Institute of Neuroscience and Mental Health The University of Melbourne Parkville Victoria Australia; ^3^ Murdoch Children's Research Institute Royal Children's Hospital, The University of Melbourne Parkville Victoria Australia; ^4^ Menzies Health Institute Queensland Griffith University Southport Queensland Australia; ^5^ Melbourne School of Population and Global Health The University of Melbourne Parkville Victoria Australia

**Keywords:** disease progression, employment, longitudinal studies, multiple sclerosis, risk factors

## Abstract

**Background and purpose:**

Understanding predictors of changes in employment status among people living with multiple sclerosis (MS) can assist health care providers to develop appropriate work retention/rehabilitation programs. We aimed to model longitudinal transitions of employment status in MS and estimate the probabilities of retaining employment status or losing or gaining employment over time in individuals with a first clinical diagnosis of central nervous system demyelination (FCD).

**Methods:**

This prospective cohort study comprised adults (aged 18–59 years) diagnosed with FCD (*n* = 237) who were followed for more than 11 years. At each review, participants were assigned to one of three states: unemployed, part‐time, or full‐time employed. A Markov multistate model was used to examine the rate of state‐to‐state transitions.

**Results:**

At the time of FCD, participants with full‐time employment had an 89% chance of being in the same state over a 1‐year period, but this decreased to 42% over the 10‐year follow‐up period. For unemployed participants, there was a 92% likelihood of remaining unemployed after 1 year, but this probability decreased to 53% over 10 years. Females, those who progressed to clinically definite MS, those with a higher relapse count, and those with a greater level of disability were at increased risk of transitioning to a deteriorated employment state. In addition, those who experienced clinically significant fatigue over the follow‐up period were less likely to gain employment after being unemployed.

**Conclusions:**

In our FCD cohort, we found a considerable rate of employment transition during the early years post‐diagnosis. Over more than a decade of follow‐up post‐FCD, we found that females and individuals with a greater disability and a higher relapse count are at higher risk of losing employment.

## INTRODUCTION

Multiple sclerosis (MS) is a complex, chronic, inflammatory and neurodegenerative disease of the central nervous system (CNS) [1]. MS typically has its onset during young adulthood when most people are actively engaged in the workforce [2]. Chronic diseases like MS, even when in an episodic phase, can affect employment outcomes and induce changes in employment status [3]. People living with MS (PwMS) report negative employment outcomes, including greater work difficulties than the general population [4]. A study conducted among 525 Australians of working age living with MS showed that 50% of the respondents missed out on work opportunities due to their health condition and 20% reported that their employment was terminated after disclosure of diagnosis [5]. Additionally, a large cross‐sectional study demonstrated that MS‐related early retirement ranged from 33% to 45% across nine European countries (*n* = 13,186) [6].

Work transitions across roles and organizations are a common feature in modern employment [3]. Therefore, employment status is dynamic and may involve leaving and re‐entering the workforce [7, 8]. Many factors including individual, situational, environmental, organizational, or socioeconomic factors can motivate a career transition [8, 9]. A diagnosis of chronic illness can affect the course of a career and motivate work transitions at multiple levels [3]. A global MS employment survey of over 12,000 people in 93 countries reported that 43% of PwMS who were unemployed had stopped working within 3 years of MS onset. This figure reached 70% 10 years after MS diagnosis [10]. Similarly, a large Swedish population‐based study (*n* = 10,137) found that PwMS had lower average levels of earnings than a reference group without MS 1 year before a formal diagnosis, and at up to 5 years post‐diagnosis [11], demonstrating that the effects of MS on employment occur early in the disease course.

Most of the previous work assessing longitudinal changes in employment among PwMS has focused on the proportion of the study cohort that is employed or unemployed at two assessment time points [7, 12, 13, 14, 15]. Although these studies successfully highlight the substantial impacts of MS on employment outcomes, they were not able to fully reflect the heterogeneity of employment trajectories among PwMS [8, 16, 17]. Notably, in our previous work from the Ausimmune Longitudinal (AusLong) study cohort, we examined trajectories of employment status using group‐based trajectory modelling (GBTM) that allowed us to define the heterogeneity of employment status, work hours, and disability support pension uptake. We were able to show that baseline characteristics (such as sex, number of comorbidities, and having children) and early MS disease factors, including higher disability level and every additional relapse, significantly predicted being in specific trajectories [8]. However, the GBTM methodology does not adequately analyze the effect of time‐varying covariates (level of disability, severity of mental health and fatigue symptoms, and number of relapses at each transition). To better understand employment transitions among PwMS, employment outcomes should not be restricted to a dichotomous variable such as employed versus unemployed [18]. An alternative is to model employment transitions, allowing people to transition up (from unemployment to part‐time or full‐time employment, and from part‐time to full‐time employment) and down (from full‐time employment to part‐time or unemployment and from part‐time employment to unemployment). This can be assessed using a multistate Markov model (MSM). This method was primarily proposed to describe the natural history of MS [19]and further applied in cost‐effectiveness analyses of therapeutic options in MS [20].The advantage of this method is that it examines individual‐level data. In addition, MSM is appropriate when the process involves transition between several well‐defined distinct states [21]. Importantly, this allows us to quantify the impact of MS‐related factors over time as it examines the factors associated with each transition irrespective of the time of the transition.

In the present study, we use an MSM to address the following research aims:

1. Examine the employment transition probabilities among a cohort of individuals with early‐onset MS who were prospectively followed for more than a decade after the first clinical diagnosis of CNS demyelination (FCD).
2. Identify predictors of employment transitions.
3. Model employment transitions over a 20‐year period and examine the impact of key predictive factors.

## PATIENTS AND METHODS

### Study population

We used data from the AusLong study, a continuation of the Ausimmune study. The Ausimmune study was a case–control study that examined the role of environmental factors on the risk of FCD among participants aged 18–59 years from four Australian regions (Brisbane, Newcastle, Geelong and Western Victoria, and Tasmania) recruited between 2003 and 2006. Since 2007, the AusLong study has prospectively followed 279 FCD cases (10‐year retention: 85%) to investigate factors associated with disease progression. Annual follow‐up has been done by nurse‐led computer‐assisted telephone interviews (CATI), where data on changes in sociodemographic and lifestyle factors over the previous 12 months were collected. Face‐to‐face reviews were conducted at baseline, 2–3 years, 5 years, and 10 years post‐FCD, at which participants underwent a detailed neurological assessment by a study neurologist, including disease status, relapse, and disability level assessment according to established criteria.

The AusLong study was approved by the Tasmanian Health and Medical Human Research Ethics Committee and by local participating research ethics committees. The study follows the ethical guidelines for human research derived from the 1964 Declaration of Helsinki. All participants provided written informed consent prior to taking part in this study.

### Outcome measures

At each CATI (annual) and face‐to‐face (baseline, 2nd/3rd year, 5th year, and 10th year) interview, participants reported their employment status using the same questions. Participants were included in the study if they had at least two observations for employment status. Employment status was ascertained using a multiple‐choice question: “Which of the following best describes your current employment status?”. The answer options were: unemployed; home duties; part‐time work/self‐employed; full‐time work/self‐employed; student; sole parent pension; disability support pension; and retired. A participant was classified as employed if they were engaged in either part‐time or full‐time employment, with all others classified as unemployed. To further ensure the work pattern, a follow‐up question was asked “Which of the following patterns do you work?”. The answer options were: regular working hours full‐time/part time; shift work full‐time/part‐time; and casual work full‐time/part‐time.

### Covariates

Baseline demographic and clinical variables identified through questionnaires/interviews included: sex, education level at the time of FCD (primary, secondary, Year 12/Higher secondary, technical, and further education [TAFE/Trade/Apprentice], university education), age at FCD, and having at least one child (yes/no). Comorbidities were assessed using the multiple‐choice question “Have you had any other illness or medical condition?” (yes/no), together with whether the comorbidity was doctor‐diagnosed (yes/no/do not know). Comorbid conditions were classified as specified in the International Statistical Classification of Diseases and Related Health Problems, Tenth Edition (ICD‐10) (Table S1). Baseline comorbidity was categorized as 0, 1–2 comorbidities, or ≥3 comorbidities. Due to our sample size and the limited number of reported comorbidities at baseline, we were unable to examine the effects of individual comorbidities due to limited power.

We assessed the following clinical characteristics annually during follow‐up: conversion to clinically definite MS (CDMS) (yes/no) based on the McDonald Criteria 2010 [22] and 2017 [23]; number of relapses since previous review; disability (Expanded Disability Status Scale (EDSS) score); and exposure to disease‐modifying therapies (DMT) (yes/no). For those who converted to CDMS and were prescribed DMT, the DMT proportion was defined as the proportion of time being on any DMT since last review. The severity of anxiety and depression symptoms were assessed using the Hospital Anxiety and Depression Scale (HADS). The seven‐item scores for each scale are summed to give the final anxiety (HADS‐A) and depression (HADS‐D) scores [24]. Consistent with the literature, a cut‐off point of 8 in each subscale was used to define clinically significant anxiety or depression [25]. To quantify the level of fatigue in the sample, the Fatigue Severity Scale (FSS), which is a nine‐item seven‐point scale (from 1 = strongly disagree to 7 = strongly agree), was utilized. An average score of ≥4.0 across the nine statements indicates a clinically significant level of fatigue [26].

### Statistical analyses

Participant characteristics were summarized with means and standard deviations (SD) for normally distributed continuous variables, and medians and interquartile ranges (IQR) for skewed continuous variables. Categorical characteristics were described with frequencies and percentages.

MSM was used to handle the recurrent employment states and the transitions between these states [21]. MSM are appropriate when the process involves individual‐level longitudinal data and transitions between several well‐defined distinct states. MSM are multilevel, discrete‐time, event history models that can be used when survival time is measured in discrete values (e.g., years to disease onset) [27]. MSM use a discrete version of the hazard function [28].

**FIGURE 1:**
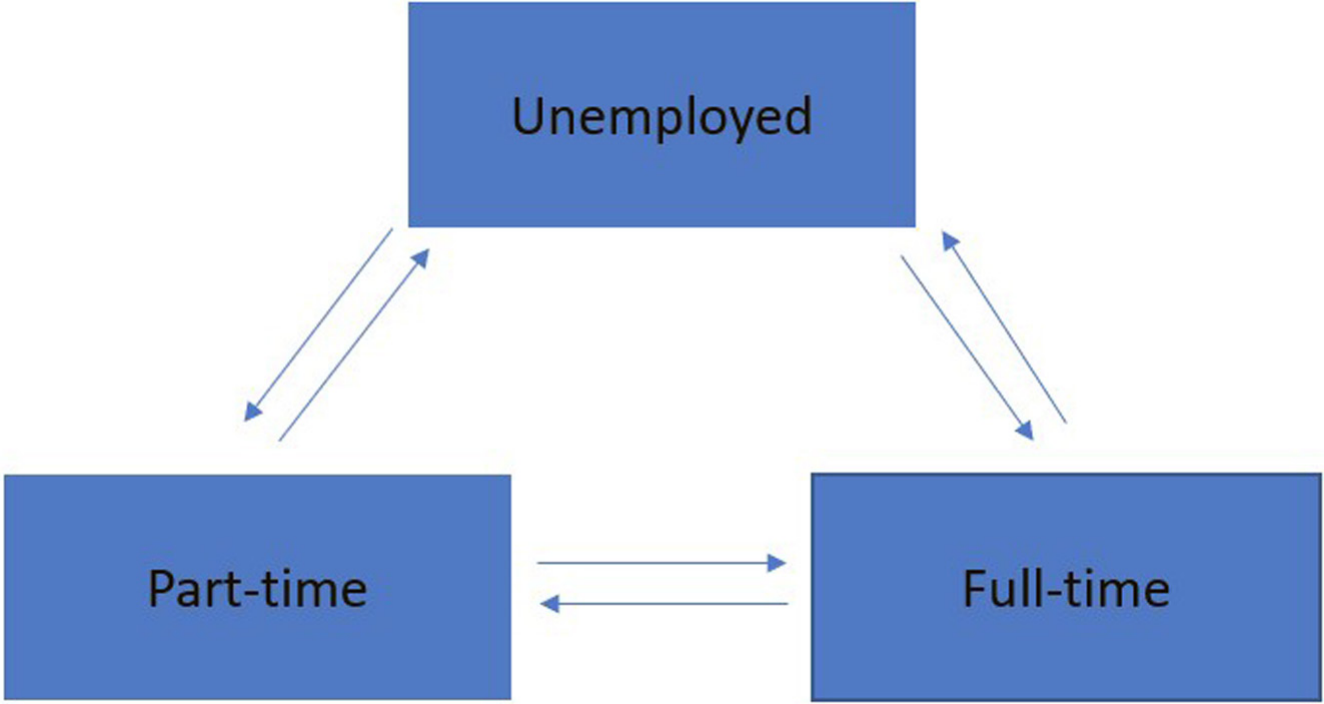
Employment transition states (box) and steps (arrow) in the AusLong cohort.

A three‐state transition model including unemployed, part‐time employment, and full‐time employment was considered in our analysis (Figure 1). First, the impact of each covariate was examined individually using a univariable transition model, establishing significant effects with a *p* ≤ 0.05. Subsequently, models were constructed incorporating two covariates focusing only on their effects on transition steps that were found to be statistically significant. We allowed for additive and interaction effects on those specific transition steps that were affected by the covariates [29]. This procedure was repeated to assess for three, four, and five, etc. covariate models, respectively, while maintaining a significance level of *p* ≤ 0.05 for all transition steps. Finally, the “lrtest.msm” function in the ‘msm’ package was employed to compare one versus two versus three versus four covariate models, etc. [27]. A global level of significance *p* ≤ 0.05 was used to determine the best employment transition model. Additionally, age was included as an a priori confounder. Based on our previous study that showed a statistically significant interaction between having children and sex on employment outcomes, we also included an interaction between having children at FCD and sex on the transition intensities. Hazard ratios (HR) and 95% confidence intervals (CI) were computed. Predicted transition probabilities were estimated up to 20 years employment transitions for different covariate characteristics. Specifically, we compared predicted transition probabilities for average age of 38 years, sex, progression to MS status, education level, and total number of comorbidities. All statistical analyses were conducted in R V.4.0.2 using the ‘msm’ package [27].

### Sensitivity analyses

We conducted sensitivity analyses to ascertain the generalizability of our findings. We performed transitional probability models within the sample for only those individuals who progressed to CDMS. In addition, considering retirement as an absorbing state, we ran the models with four distinct states.

## RESULTS

Of 279 participants at baseline, 237 (84.9%) had at least two observations and were included in this study. Participant characteristics (*n* = 237) are summarized in Table 1. Of the study participants, 68.3% (*n* = 162) progressed to CDMS at 5 years and 83.5% (*n* = 198) at 10 years post‐FCD. Fourteen participants (5.9%) had progressive‐onset MS.

**TABLE 1:** Demographic and clinical characteristics of AusLong participants at baseline and during follow‐up included in the analyses (*N* = 237).

Variable	Sample (*N* = 237)
Female sex, *n* (%)	184 (77.6)
Age at FCD (years), mean (SD)	37.8 (9.4)
Education level at FCD, *n* (%)	
Up to Higher secondary/Year 12	105 (44.3)
TAFE/Trade/Apprentice	71 (30.0)
University	61 (25.7)
Have ≥1 child at FCD,^a^ *n* (%)	156 (66.7)
Comorbidities at FCD, *n* (%)	
0	93 (39.2)
1–2	115 (48.6)
≥3	29 (12.2)
CDMS by 10‐year review, *n* (%)	198 (83.5)
Average years from FCD to CDMS, median (IQR)	0.9 (0.3–2.5)
Exposure to DMT, *n* (%)	157 (66.2)
Follow‐up duration from FCD, mean (SD)	11.4 (2.3)
Number of relapses since previous review, median (IQR)	1 (1–2)

Employment‐related characteristics	Baseline (*N* = 237)	10–year review^a^ (*N* = 224)
Employment status, *n* (%)		
Unemployed	56 (23.6)	75 (33.5)
Part‐time employment	67 (28.3)	79 (35.3)
Full‐time employment	114 (48.1)	70 (31.2)

Abbreviations: CDMS, clinically definite multiple sclerosis; DMT, disease‐modifying treatment; FCD, first clinical diagnosis of central nervous system demyelination; IQR, interquartile range; SD, standard deviation; TAFE, technical and further education.

^a^ Data for three participants were missing.

### Aim 1: Employment transition probabilities

The total number of observed employment states at each review in the AusLong cohort (*n* = 237) over the 10‐year follow‐up period is reported in Table 2. Table 3 provides the frequencies of pairs of consecutive observed states for employment status (maintenance and transitions) over the 10‐year follow‐up period. We counted the number of times participants remained in the same state of employment or moved to another state. Across the whole dataset (all participants over the 10‐year follow‐up), there were 2028 recorded observations, of which 495 (24.4%) observations of participants remaining unemployed from one assessment to the next. Notably, the most common state was remaining in full‐time employment, for which there were 665 (32.8%) observations.

**TABLE 2:** Number of observed employment transition states over the follow‐up period.

Review, year	Y1	Y2	Y3	Y4	Y5	Y6	Y7	Y8	Y9	Y10
Observed, *n*	235	234	235	236	236	230	224	214	206	224

**TABLE 3:** Observations of employment status maintenance (having the same employment status) and transition (changing employment status) between annual follow‐up assessments among people living with multiple sclerosis in the AusLong cohort (*N* = 237) over a 10‐year period.

Employment status at the index review, *n* (%)	Employment status at next review		
	Unemployed	Part‐time	Full‐time
Unemployed	495 (24.4)	41 (2.0)	12 (0.6)
Part‐time	48 (2.4)	599 (29.5)	58 (2.9)
Full‐time	32 (1.6)	78 (3.8)	665 (32.8)

Table 4 presents the estimated transitional probabilities between each employment status over a 1‐year, 5‐year, and 10‐year period post‐FCD, and also shows the probability of staying in a particular employment state. We found that a participant in full‐time employment at FCD had an 89.0% chance of being in the same state at the 1‐year follow‐up. This decreased to 59.8% and 42.4% at the 5‐year and 10‐year follow‐ups, respectively. Similar trends were observed for individuals who worked part‐time. In contrast, a participant who was unemployed at FCD was found to have a 92.0% chance of remaining unemployed at the 1‐year follow‐up, though this probability decreased to 68.9% and 53.2% at the 5‐year and 10‐year follow‐ups, respectively.

**TABLE 4:** Estimated 1‐year, 5‐year, and 10‐year transition probabilities (95% CIs) within and to each employment state among people living with multiple sclerosis in the AusLong cohort (*N* = 237).

Employment status at FCD	Estimated 1‐year transition probabilities, % (95% CI)			Estimated 5‐year transition probabilities, % (95% CI)			Estimated 10‐year transition probabilities, % (95% CI)		
	Unemployed	Part‐time	Full‐time	Unemployed	Part‐time	Full‐time	Unemployed	Part‐time	Full‐time
Unemployed (*n* = 56)	92.0 (89.4–93.8)	6.1 (4.5–8.1)	2.0 (1.2–3.4)	68.9 (61.7–74.7)	21.5 (16.7–27.6)	9.5 (7.0–13.9)	53.2 (44.3–61.0)	30.1 (24.3–36.8)	16.7 (12.9–22.3)
Part‐time (*n* = 67)	5.4 (4.1–7.1)	88.2 (85.9–90.1)	6.4 (5.0–8.1)	19.8 (15.65–24.9)	59.4 (54.0–65.1)	20.8 (17.0–25.4)	28.5 (23.2–34.1)	44.9 (38.8–50.9)	26.6 (21.5–32.3)
Full‐time (*n* = 114)	3.4 (2.5–4.7)	7.6 (6.2–9.6)	89.0 (86.6–90.7)	14.6 (11.5–18.7)	25.6 (21.4–30.5)	59.8 (54.0–64.9)	23.9 (19.3–29.4)	33.7 (28.5–38.6)	42.4 (36.0–49.1)

Abbreviations: CI, confidence interval; FCD, first clinical diagnosis of central nervous system demyelination.

In terms of employment transitions, we found that the chances of participants with full‐time employment status at FCD transitioning to part‐time employment increased markedly over the follow‐up period. At the 1‐year follow‐up, there was a 7.6% chance that they would transition to part‐time employment. This increased to 25.6% and 33.7% at the 5‐year and 10‐year follow‐ups, respectively. Conversely, participants who were working part‐time at FCD were found to have fairly similar probability of moving to either full‐time employment or unemployment at the end of the 1‐year (unemployment: 5.4%; full‐time employment: 6.4%), 5‐year (unemployment: 19.8%; full‐time employment: 20.8%), and 10‐year (unemployment: 28.5%; full‐time employment: 26.6%) follow‐ups. We also found that unemployed participants had a higher probability of moving to a part‐time employment state compared to full‐time at the 1‐year (6.1% vs. 2.0%), 5‐year (21.5% vs. 9.5%), and 10‐year (30.1% vs. 16.7%) follow‐ups.

### Aim 2: Factors that are predictive of employment transitions

Table 5 presents the results of univariable MSM models evaluating the effect of baseline covariates (sex, educational level, having children, and number of comorbidities at FCD) and time‐varying covariates (age, progression to CDMS, disability level [measured by EDSS], relapse count, having clinically significant fatigue, depression, and anxiety) on the HR of transition between employment states over the follow‐up period. The interaction model including sex and having children at FCD was found to be significant in transitions between part‐time to both unemployed and full‐time states and thus the interaction term was included in the final multivariable model. This did not change by adjusting for confounding in the multivariable regression model (Table S2).

**TABLE 5:** Results from univariable multistate models for losing and gaining employment following a first clinical diagnosis of central CNS demyelination in the AusLong cohort (*N* = 237).

Covariate	Decreasing or losing employment			Increasing or gaining employment		
	Full‐time to part‐time	Full‐time to unemployed	Part‐time to unemployed	Unemployed to part‐time	Unemployed to full‐time	Part‐time to full‐time
	HR (95% CI)	HR (95% CI)	HR (95% CI)	HR (95% CI)	HR (95% CI)	HR (95% CI)
Age	1.03 (0.96–1.11)	0.99 (0.88–1.11)	1.01 (0.92–1.11)	0.93 (0.84–1.03)	0.85 (0.70–1.04)	1.01 (0.92–1.10)
Sex						
Male	Ref (1.00)	Ref (1.00)	Ref (1.00)	Ref (1.00)	Ref (1.00)	Ref (1.00)
Female	3.57 (1.93–6.61)*	0.95 (0.47–1.92)	0.86 (0.27–2.77)	3.69 (0.89–15.28)	2.08 (0.27–16.13)	0.27 (0.14–0.54)*
Have ≥1 child at FCD						
No	Ref (1.00)	Ref (1.00)	Ref (1.00)	Ref (1.00)	Ref (1.00)	Ref (1.00)
Yes	0.63 (0.40–0.99)*	1.14 (0.55–2.35)	0.64 (0.36–1.16)	0.69 (0.36–1.33)	0.45 (0.14–1.47)	0.49 (0.29–0.83)*
Education level at FCD						
High school/Year 12	Ref (1.00)	Ref (1.00)	Ref (1.00)	Ref (1.00)	Ref (1.00)	Ref (1.00)
TAFE/Trade/Apprentice	0.89 (0.50–1.58)	0.58 (0.24–1.39)	1.30 (0.66–2.55)	1.17 (0.57–2.39)	0.81 (0.15–4.43)	0.88 (0.46–1.66)
University	1.27 (0.76–2.13)	0.64 (0.28–1.50)	0.95 (0.47–1.91)	2.27 (1.05–4.91)*	6.12 (1.73–21.68)*	0.88 (0.48–1.62)
Number of comorbidities at FCD						
0	Ref (1.00)	Ref (1.00)	Ref (1.00)	Ref (1.00)	Ref (1.00)	Ref (1.00)
1–2	0.93 (0.58–1.49)	0.65 (0.29–1.46)	1.80 (0.93–3.49)	0.73 (0.38–1.41)	1.83 (0.46–7.32)	1.01 (0.59–1.74)
≥3	1.03 (0.47–2.26)	2.50 (1.02–6.12)*	2.42 (1.01–5.83)*	0.50 (0.19–1.34)	2.02 (0.41–10.00)	0.98 (0.40–2.40)
Progression to CDMS						
No	Ref (1.00)	Ref (1.00)	Ref (1.00)	Ref (1.00)	Ref (1.00)	Ref (1.00)
Yes	1.72 (1.03–2.89)*	2.99 (1.53–7.77)*	1.29 (0.63–2.67)	0.78 (0.38–1.59)	0.35 (0.11–1.11)	0.78 (0.44–1.39)
EDSS increase per point	1.23 (1.05–1.44)*	1.50 (1.21–1.86)*	1.23 (1.01–1.50)*	0.84 (0.70–1.03)	0.69 (0.46–1.04)	0.89 (0.72–1.10)
Number of relapses	1.04 (0.96–1.12)	1.10 (1.01–1.21)*	1.10 (1.02–1.18)*	1.04 (0.98–1.10)	1.05 (0.95–1.16)	1.00 (0.90–1.10)
Clinically significant anxiety (HADS Anxiety≥8)						
No	Ref (1.00)	Ref (1.00)	Ref (1.00)	Ref (1.00)	Ref (1.00)	Ref (1.00)
Yes	0.81 (0.39–1.69)	1.32 (0.51–3.42)	0.90 (0.40–2.01)	0.59 (0.25–1.41)	0.69 (0.15–3.15)	1.52 (0.82–2.83)
Clinically significant depression (HADS Depression≥8)						
No	Ref (1.00)	Ref (1.00)	Ref (1.00)	Ref (1.00)	Ref (1.00)	Ref (1.00)
Yes	1.59 (0.69–3.65)	1.27 (0.30–5.31)	0.90 (0.28–2.91)	0.48 (0.17–1.36)	0.41 (0.05–3.15)	0.74 (0.23–2.36)
Clinically significant fatigue (FSS≥4)						
No	Ref (1.00)	Ref (1.00)	Ref (1.00)	Ref (1.00)	Ref (1.00)	Ref (1.00)
Yes	1.39 (0.84–2.32)	1.58 (0.73–3.42)	0.87 (0.49–1.55)	0.71 (0.35–1.46)	0.16 (0.05–0.52)*	0.85 (0.51–1.43)

*Bold type denotes statistical significance (*p* ≤ 0.05).

Abbreviations: CDMS, clinically definite multiple sclerosis; CI, confidence interval; EDSS, Expanded Disability Status Scale; FCD, first clinical diagnosis of central nervous system demyelination; FFS, Fatigue Severity Scale; HADS, Hospital Anxiety and Depression Scale; HR, hazard ratio; Ref, reference; TAFE, technical and further education.

#### Predictors of reduced employment or losing employment

The multivariable MSM (Table 6) demonstrates that females were 2.7 times as likely to transition from full‐time to part‐time employment compared to males. For every additional EDSS point accrued during follow‐up, the hazard of moving to a deteriorated employment state (part‐time or unemployed) increased by 17%–57%. Similarly, every additional relapse was associated with a 11% greater risk of moving from part‐time employment to unemployment. Further, progression to CDMS was associated with a 4 times higher risk of moving from full‐time employment to unemployment (HR = 4.03; 95% CI = 1.35–12.05).

**TABLE 6:** Results from multivariable multistate models for losing and gaining employment following a first clinical diagnosis of CNS demyelination in the AusLong cohort (*N* = 237).

Covariate	Decreasing or losing employment			Increasing or gaining employment		
	Full‐time to part‐time	Full‐time to unemployed	Part‐time to unemployed	Unemployed to part‐time	Unemployed to full‐time	Part‐time to full‐time
	HR (95% CI)	HR (95% CI)	HR (95% CI)	HR (95% CI)	HR (95% CI)	HR (95% CI)
Sex						
Male	Ref (1.00)	Ref (1.00)	Ref (1.00)	Ref (1.00)	Ref (1.00)	Ref (1.00)
Female	**2.74 (1.40–5.34)** *	–	–	–	–	0.46 (0.17–1.24)
Have ≥1 child at FCD						
No	Ref (1.00)	Ref (1.00)	Ref (1.00)	Ref (1.00)	Ref (1.00)	Ref (1.00)
Yes	0.63 (0.38–0.1.04)	–	–	–	–	2.27 (0.65–7.91)
Education level at FCD						
High school/Year 12	Ref (1.00)	Ref (1.00)	Ref (1.00)	Ref (1.00)	Ref (1.00)	Ref (1.00)
TAFE/Trade/Apprentice	–	–	–	1.08 (0.52–2.24)	0.49 (0.05–4.44)	–
University	–	–	–	**2.35 (1.08–5.12)** *	**6.24 (1.74–22.42)** *	–
Number of comorbidities at FCD						
0	Ref (1.00)	Ref (1.00)	Ref (1.00)	Ref (1.00)	Ref (1.00)	Ref (1.00)
1–2	–	0.61 (0.26–1.42)	1.86 (0.89–3.88)	–	–	–
≥3	–	2.48 (0.97–6.36)	2.50 (0.89–7.00)	–	–	–
Progression to CDMS						
No	Ref (1.00)	Ref (1.00)	Ref (1.00)	Ref (1.00)	Ref (1.00)	Ref (1.00)
Yes	1.57 (0.85–2.89)	**4.03 (1.35–12.05)** *	–	–	–	–
EDSS increase per point	1.17 (0.98–1.42)	**1.57 (1.24–1.99)** *	1.18 (0.92–1.52)	–	–	–
Number of relapses	–	1.04 (0.93–1.16)	**1.11 (1.02–1.21)** *	–	–	–
Clinically significant fatigue (FSS≥4)						
No	Ref (1.00)	Ref (1.00)	Ref (1.00)	Ref (1.00)	Ref (1.00)	Ref (1.00)
Yes	–	–	–	–	**0.18 (0.05–0.69)** *	–

*Note*: Multivariable models were adjusted for age.

*Bold type denotes statistical significance (*p* ≤ 0.05). Abbreviations: CDMS, clinically definite multiple sclerosis; CI, confidence interval: EDSS, Expanded Disability Status Scale; FCD, first clinical diagnosis of central nervous system demyelination; FFS, Fatigue Severity Scale; HR, hazard ratio; Ref, reference; TAFE, technical and further education.

#### Predictors of increased employment or gaining employment

The multivariable MSM (Table 6) demonstrates that having a university education was strongly associated with a 2 to 6 times greater likelihood of gaining employment (to part‐time and full‐time employment, respectively) following unemployment. None of the MS‐related factors were found to be a significant predictor of gaining employment in our cohort. However, experiencing clinically significant fatigue during follow‐up was associated with an 82% decreased risk of gaining full‐time employment (HR = 0.18; 95% CI = 0.05–0.69).

In a univariable model, the subset of the AusLong cohort who progressed to CDMS, having used a DMT for ≥90% of time since the last review, was a significant predictor for transition from part‐time to full‐time employment (HR = 2.03; 95% CI = 1.75–3.83). However, this association did not remain significant in the multivariable model (HR = 1.72; 95% CI = 0.85–3.47).

### Aim 3: Model of employment transitions

**FIGURE 2:**
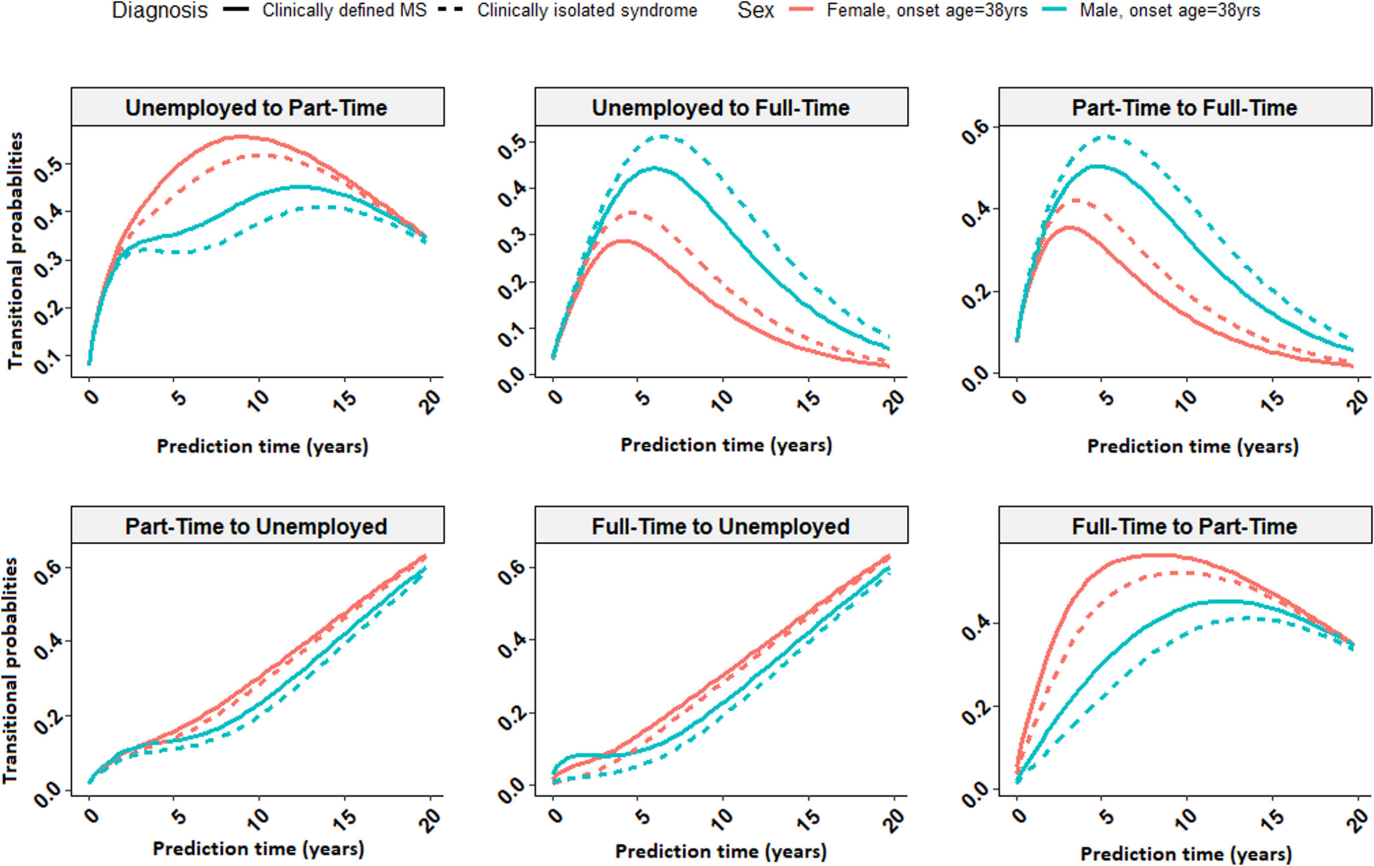
Predicted employment transitions over a 20‐year period for a participant who is 38 years old at the time of first clinical diagnosis of central nervous system demyelination based on the fitted final multivariable model by sex and progression to clinically definite multiple sclerosis.

Using the final fitted model, Figure 2 shows the predicted employment transitions over a 20‐year period post‐FCD for an individual at an average age at FCD (38 years old) based on sex and progression to CDMS. In terms of employment loss, the probability of moving to unemployment increased with increasing disease duration. In contrast, the probability of gaining full‐time employment only increased in the first few years following FCD and then gradually decreased. The probability of losing employment was constantly higher in females and those who progressed to CDMS compared to males and those who did not progress to CDMS at each time point (Figure 2). The predicted model of employment transitions based on education level and number of comorbidities at baseline are presented in Figures S1 and S2.

### Sensitivity analyses

Excluding individuals who remained as FCD and did not progress to CDMS did not materially change the estimates for the predictors of employment transitions except for having more than three comorbidities at baseline, which were associated with a 2.7 to 4.5 times increased risk of moving to unemployed state from full‐time and part‐time employment, respectively (Table S3 and Figure S2). Furthermore, as regards having a retired state as the fourth state, we were not able to conduct sensitivity analysis for the predictors of employment transitions due to limited sample size in the retired state (Table S4).

## DISCUSSION

In this study we have described the rate of employment transitions in a cohort of individuals with a first clinical diagnosis of CNS demyelination over a decade in Australia. We utilized a novel approach, multistate Markov models, to estimate the short‐ and long‐term employment transition probabilities in our cohort. Our study confirmed several demographic factors (including sex and education level) that are known to be predictive of employment transitions. Our study also extended previous findings by identifying significant time‐dependent patterns in the predictive strength of clinical and MS‐specific factors (including progression to CDMS, disability level, number of relapses, and fatigue) related to employment transitions among PwMS. Specifically, differentiating between predictors of losing and gaining employment, and assessing the degree to which these predictors impact employment transition in a time‐dependent manner, provides significant insights into the effect of a first demyelinating event and early MS on employment outcomes.

The AusLong cohort provides a unique opportunity to assess employment transitions in the first decade after an episode of CNS demyelination. As 83.5% of the AusLong cohort progressed to CDMS by the 10‐year review, our analysis presents findings that are of importance to the MS community. We found that the probability of transition from unemployment or full‐time employment to part‐time employment increased from 6.1%–7.6% at the 1‐year review to 30.1%–33.7% at the 10‐year review, highlighting the fact that PwMS are more likely to work part‐time as MS progresses [8, 12, 13, 14]. Furthermore, over the follow‐up period, the probability of becoming unemployed for part‐time (1‐year: 5.4%; 10‐year: 28.5%) and full‐time (1‐year: 3.4%; 10‐year: 23.9%) employment states consistently increased. This is in line with the findings of previous prospective studies which reported increased probability of unemployment after onset of MS [8, 14, 30]. It is noteworthy that previous studies used different outcomes at different time points which made it difficult to compare our findings.

We found several demographic and clinical factors that predicted subsequent employment transitions in the study cohort, particularly sex and education level. We found that females had an increased risk of transitioning from full‐time to part‐time employment. This is consistent with our previous work in this cohort, which shows that females were at increased risk of being employed part‐time compared to males [8]. We also found that having a university degree was associated with a greater chance of re‐entering part‐time and full‐time employment following unemployment. The current finding may relate to university education and having a higher chance of being successful with job applications. This result also highlights the protective effects of greater education attainment that have been documented by previous studies [7, 31, 32]. Conversely, this may reflect the greater impact of MS on lower skill level employment (such as manual labor) [4]. Consistent with previous studies, among the sample who progressed to CDMS, we found that having ≥3 comorbidities at MS onset was associated with greater risk of unemployment [33, 34].

We found that MS‐related factors, including disability level, the number of relapses, and fatigue, were significantly associated with the risk of losing employment. For every additional EDSS point accrued during follow‐up, the risk of transition from full‐time employment to unemployment increased by 57%. This agrees with two recent systematic reviews (Kavaliunas et al., 2022 [35], Gerhard et al., 2020 [31]) that highlight the considerable effect of physical disability on economic participation among PwMS. Likewise, every additional relapse during follow‐up was associated with a 4% and 11% increased risk of moving to unemployment from full‐time employment or part‐time employment, respectively. Although relapse is amongst the disease‐specific factors suggested to contribute to employment loss [14], results from previous cohort studies are inconsistent, with some finding relapse rate a significant predictor of part‐time employment [8] but others not finding an association [12]. The effect of DMT use in this cohort may have significantly reduced the number of relapses, but the association between relapses and losing employment despite therapy remained significant. We found that fatigue was associated with a reduced transition from unemployed to part‐time employed. This is in line with previous research studies where fatigue was identified as a contributor to higher work impairment [33], diminished job retention [36], employment change [18], and being unemployed [31].

In our model of the probability of employment transition over a 20‐year period in a young adult aged 38 years at FCD, we found that the probability of moving from full‐time to part‐time employment increased sharply in the first 5 years post‐FCD and then gradually decreased. Similarly, the probability of returning to full‐time employment began to decrease after 5 years post‐FCD. Females and those who progressed to CDMS were less likely to return to full‐time employment compared with males and those who remained in the clinically isolated syndrome (CIS) state. This agrees with previous work showing that the median time to becoming unemployed or working part‐time due to MS was approximately 6 years [12].

Our study highlights the necessity of identifying risk factors that may account for early employment loss and suggests introducing early intervention aimed at addressing employment concerns before the onset of disease progression. These findings underscore the necessity of evidence‐based rehabilitation and employment support programs at the time of MS diagnosis [37]. The first Cochrane review by Khan and colleagues in 2009 found inconclusive evidence to support vocational rehabilitation for PwMS; however, it underlined the importance of timely interventions to deal with work disability, workplace accommodation, and enhancing employer's knowledge and attitudes toward MS disease [38]. Any reasonable adjustment in the workplace or pattern such as adopting flexible hours, working from home, giving additional time for completing tasks, and use of equipment/assistive technological resources particularly in participants with greater disability and more severe disease course have been reported as common work accommodations [39]. However, there is still insufficient evidence about how to design and resource a sustainable vocational rehabilitation service and the cost–benefit and effectiveness of rehabilitation models to support work retention in PwMS considering the varied social and welfare systems in different countries [40].

### Strengths and limitations

A key strength of this study was the collection of comprehensive time‐varying clinical factors prospectively from FCD/CIS and early MS onset over a long‐term follow‐up period. Further, using a robust statistical analysis method allowed us to determine the magnitude of the impact of demographic and clinical factors on employment transitions. This enabled us to observe a more realistic pattern of employment change and made our estimates generalizable to cohorts with similar characteristics. Our study has three key limitations. First, in Australia, the social welfare and pension structure, and the National Disability Insurance Scheme (NDIS), may affect employment‐related decisions among PwMS. Hence, our findings may not be generalizable to countries with different sociodemographic or welfare systems. Second, there are other factors, including personal (cognition, pain) and work‐related factors (job type, workplace, and public policy), that may affect the employment transitions in our sample which were not examined in this study. Third, this study was conducted during a time in which high‐efficacy DMTs were not available for most of the cohort. Contemporary studies with higher‐efficacy therapies may produce different results.

## CONCLUSIONS

We documented the rate of employment transitions in a cohort of individuals with a first episode of CNS demyelination followed over a long period. We highlighted the fact that employment outcomes cannot be restricted to an employed/unemployed dichotomy. We have shown that female sex, progression to CDMS, more rapid disability progression, and a greater number of relapses significantly increase the risk of a negative employment transition. Clinically significant fatigue was found to significantly increase the risk of not re‐entering the workforce, while having a university degree significantly increased the chances of transitioning from unemployed to part‐time and full‐time employment. The association between MS‐specific factors (disability level in particular) and employment outcomes demonstrated here and elsewhere underscore the potential of introducing employment outcomes as an appropriate proxy for MS progression in future clinical trials and registry studies [35]. Understanding the multidimensional patterns of early and time‐dependent predictors of employment transitions is important to optimally target work‐retention programs tailored to these factors among PwMS who may need additional work‐related assistance as early as possible during the disease course in order to remain longer in the labor force.

## FUNDING INFORMATION

Financial support for this research was provided by grants from the National Multiple Sclerosis Society of the United States of America (award RG3364A1/2) and the National Health and Medical Research Council of Australia (APP316901, 224215, and 1127819).

## CONFLICT OF INTEREST STATEMENT

None declared.

## PATIENT CONSENT

Patient consent obtained.

## Supporting information


**Table S1** Categories of comorbidities included in the AusLong cohort.
**TABLE S2** Analysis of models for investigating the interaction between sex and having a child at first clinical diagnosis of central nervous system demyelination (FCD) on losing and gaining employment in the AusLong cohort.
**TABLE S3** Sensitivity analysis of multivariable multistate models for losing and gaining employment among individuals who progressed to clinically definite multiple sclerosis (CDMS) in the AusLong cohort (*n* = 198).
**TABLE S4** Observations of employment status maintenance (having the same employment status) and transition (changing employment status) between annual follow‐up assessments among people living with multiple sclerosis (PwMS) in the AusLong cohort (*n* = 237) over a 10‐year period considering the retired state as an absorbing state.
**FIGURE S1** Predicted employment transitions over a 20‐year period for a participant who is 38 years old at the time of first clinical diagnosis of central nervous system demyelination (FCD) based on the fitted final multivariable model by education level and progression to clinically definite multiple sclerosis (CDMS).
**FIGURE S2** Predicted employment transitions over a 20‐year period for a participant who is 38 years old at the time of first clinical diagnosis of central nervous system demyelination (FCD) based on the fitted final multivariable model by number of total comorbidities at baseline and progression to clinically definite multiple sclerosis (CDMS).

## Data Availability

The data that support the findings of this study are available on request from the corresponding author. The data are not publicly available due to privacy or ethical restrictions.
