# Supplementary material for: Changes in employment status over time in multiple sclerosis following a first episode of central nervous system demyelination, a Markov multistate model study
Source: Eur J Neurol. 2023 Aug 13;31(1):e16016. doi: 10.1111/ene.16016 (PMC11235915; doi:10.1111/ene.16016)
Supplement: Supplementary file 1 — Table S1 Categories of comorbidities included in the AusLong cohort. TABLE S2 Analysis of models for investigating the interaction between sex and having a child at first clinical diagnosis of central nervous system demyelination (FCD) on losing and gaining employment in the AusLong cohort. TABLE S3 Sensitivity analysis of multivariable multistate models for losing and gaining employment among individuals who progressed to clinically definite multiple sclerosis (CDMS) in the AusLong cohort (n = 198). TABLE S4 Observations of employment status maintenance (having the same employment status) and transition (changing employment status) between annual follow‐up assessments among people living with multiple sclerosis (PwMS) in the AusLong cohort (n = 237) over a 10‐year period considering the retired state as an absorbing state. FIGURE S1 Predicted employment transitions over a 20‐year period for a participant who is 38 years old at the time of first clinical diagnosis of central nervous system demyelination (FCD) based on the fitted final multivariable model by education level and progression to clinically definite multiple sclerosis (CDMS). FIGURE S2 Predicted employment transitions over a 20‐year period for a participant who is 38 years old at the time of first clinical diagnosis of central nervous system demyelination (FCD) based on the fitted final multivariable model by number of total comorbidities at baseline and progression to clinically definite multiple sclerosis (CDMS). [file ENE-31-e16016-s001.docx]

**Supplementary Table 1:** Categories of comorbidities included in the AusLong cohort

| **ICD-10 categories of comorbidity** |
| --- |
| **Diseases of the blood** |
| Anaemia |
| **Mental and behavioural disorders** |
| Mood [affective] disorders |
| Neurotic, stress-related and somatoform disorders |
| **Diseases of the musculoskeletal system and connective tissue** |
| Osteoporosis |
| Osteoarthritis |
| Rheumatoid arthritis |
| Psoriatic arthropathies |
| Systemic connective tissue disorders |
| Gout |
| Dorsopathies |
| Soft tissue disorders |
| **Diseases of the circulatory system** |
| Essential hypertension |
| Cerebrovascular diseases |
| Ischaemic heart diseases |
| Other forms of heart disease |
| Peripheral vascular diseases |
| **Endocrine, nutritional and metabolic diseases** |
| Disorders of lipoprotein metabolism and other lipidaemias |
| Disorders of thyroid gland |
| Diabetes mellitus |
| Polycystic ovarian syndrome |
| Haemochromatosis |
| Adrenogenital disorders |
| **Diseases of the digestive system** |
| Gastro-oesophageal reflux disease |
| Hernia |
| Irritable Bowel Syndrome |
| Inflammatory Bowel Disease |
| **Diseases of the respiratory system** |
| Asthma |
| Other chronic obstructive pulmonary disease |
| Chronic sinusitis |
| **Diseases of the nervous system** |
| Epilepsy |
| Migraine |
| Sleep apnoea |
| Myasthenia gravis |
| Parkinson disease |
| **Diseases of the genitourinary system** |
| Chronic renal disease |
| Noninflammatory disorders of female genital tract |
| **Neoplasms** |
| Malignant neoplasms |
| Other neoplasms |
| **Diseases of the skin and subcutaneous tissue** |
| Psoriasis |
| Dermatitis |
| Bullous disorders |
| **Diseases of the blood and blood forming organs and certain disorders involving the immune mechanism** |
| Sarcoidosis |
| **Diseases of the eye and adnexa** |
| **Congenital malformations, deformations and chromosomal abnormalities** |
| **Allergies** |

**Supplementary Table 2.** Analysis of models for investigating the interaction between sex and having child at FCD on losing and gaining employment in the AusLong cohort

| Covariates | Decreasing or losing employment | | | Increasing or gaining employment | | |
| --- | --- | --- | --- | --- | --- | --- |
|  | **Full-time to part-time** | **Full-time to unemployed** | **Part-time to unemployed** | **Unemployed to part-time** | **Unemployed to full-time** | **Part-time to full-time** |
|  | Hazard Ratio (95%CI) | Hazard Ratio (95%CI) | Hazard Ratio (95%CI) | Hazard Ratio (95%CI) | Hazard Ratio (95%CI) | Hazard Ratio (95%CI) |
| Sex |  |  |  |  |  |  |
| Male | Ref (1.00) | Ref (1.00) | Ref (1.00) | Ref (1.00) | Ref (1.00) | Ref (1.00) |
| Female | 3.57(1.93‒6.61)* | 0.95(0.47‒1.92) | 0.86(0.27‒2.77) | 3.69(0.89‒15.28) | 2.08(0.27‒16.13) | 0.27(0.14‒0.54)* |
| Have ≥1 child at FCD |  |  |  |  |  |  |
| No | Ref (1.00) | Ref (1.00) | Ref (1.00) | Ref (1.00) | Ref (1.00) | Ref (1.00) |
| Yes | 0.63(0.40‒0.99)* | 1.14(0.55‒2.35) | 0.64(0.36‒1.16) | 0.69(0.36‒1.33) | 0.45(0.14‒1.47) | 0.49(0.29‒0.83)* |
| Interaction |  |  |  |  |  |  |
| Sex* Have ≥1 child at FCD | 1.98(0.76-5.15) | 1.49(0.34-6.56) | 0.11(0.02-0.46)* | 4.68(0.95-22.96) | 0.68(0.13-3.65) | 0.21(0.08-0.59)* |

* P value less than 0.05

**Supplementary Table 3.** Sensitivity analysis of multivariable multistate models for losing and gaining employment among individuals who progressed to CDMS in the AusLong Cohort (n=198)

| Covariates | Decreasing or losing employment | | | Increasing or gaining employment | | |
| --- | --- | --- | --- | --- | --- | --- |
|  | **Full-time to part-time** | **Full-time to unemployed** | **Part-time to unemployed** | **Unemployed to part-time** | **Unemployed to full-time** | **Part-time to full-time** |
|  | Hazard Ratio (95%CI) | Hazard Ratio (95%CI) | Hazard Ratio (95%CI) | Hazard Ratio (95%CI) | Hazard Ratio (95%CI) | Hazard Ratio (95%CI) |
| Sex |  |  |  |  |  |  |
| Male | Ref (1.00) | Ref (1.00) | Ref (1.00) | Ref (1.00) | Ref (1.00) | Ref (1.00) |
| Female | **3.66(1.54‒8.70)*** | - | - | - | - | 0.57(0.17‒1.96) |
| Have ≥1 child at FCD |  |  |  |  |  |  |
| No | Ref (1.00) | Ref (1.00) | Ref (1.00) | Ref (1.00) | Ref (1.00) | Ref (1.00) |
| Yes | 0.70(0.40‒0.23) | - | - | - | - | 2.02 (0.33‒12.36) |
| Education level at FCD |  |  |  |  |  |  |
| High school / year 12 | Ref (1.00) | Ref (1.00) | Ref (1.00) | Ref (1.00) | Ref (1.00) | Ref (1.00) |
| TAFE/Trade/Apprentice | - | - | - | 0.91(0.37‒2.26) | 0.66(0.07‒6.45) | - |
| University | - | - | - | **3.09(1.36‒7.03)*** | 2.56(0.42‒15.69) | - |
| Number of comorbidities at FCD |  |  |  |  |  |  |
| 0 | Ref (1.00) | Ref (1.00) | Ref (1.00) | Ref (1.00) | Ref (1.00) | Ref (1.00) |
| 1-2 | - | 0.55(0.22**‒**1.35) | **2.88(1.11‒7.43)*** | - | - | - |
| ≥3 |  | **2.74(1.03‒7.28)** | **4.45(1.30‒15.17)*** |  |  |  |
| EDSS increase per point | 1.12(0.90‒1.39) | **1.51(1.15 ‒1.97)*** | 1.23(0.93‒1.63) | - | - | - |
| Number of relapses | - | 0.91(0.76‒1.08) | **1.17(1.05‒1.29)*** | - | - | - |
| More than 90% on DMT |  |  |  |  |  |  |
| No | Ref (1.00) | Ref (1.00) | Ref (1.00) | Ref (1.00) | Ref (1.00) | Ref (1.00) |
| Yes | - | - | **-** | - | - | 1.81(0.93‒3.52) |
| Clinically significant fatigue (FSS≥4) |  |  |  |  |  |  |
| No | Ref (1.00) | Ref (1.00) | Ref (1.00) | Ref (1.00) | Ref (1.00) | Ref (1.00) |
| Yes | - | - | - | - | **0.18(0.03‒0.97)*** | - |

Multivariable models were adjusted for age. Predictors that were not significant for each transition in the univariable models were not included in the multivariable models and is reported by (-). * P value less than 0.05

**Supplementary Table 4.** Observations of employment status maintenance (having the same employment status) and transition (changing employment status) between annual follow-up assessments among PwMS in the AusLong Cohort (n=237) over a 10-year period considering the retired state as an absorbing state.

|  | **Employment status at next review** | | | |
| --- | --- | --- | --- | --- |
| **Employment status at the index review** | **Unemployed** | **Part-time** | **Full-time** | **Retired** |
| Unemployed | 439 | 40 | 12 | 7 |
| Part-time | 42 | 595 | 58 | 7 |
| Full-time | 26 | 78 | 665 | 6 |
| Retired | 0 | 0 | 0 | 43 |


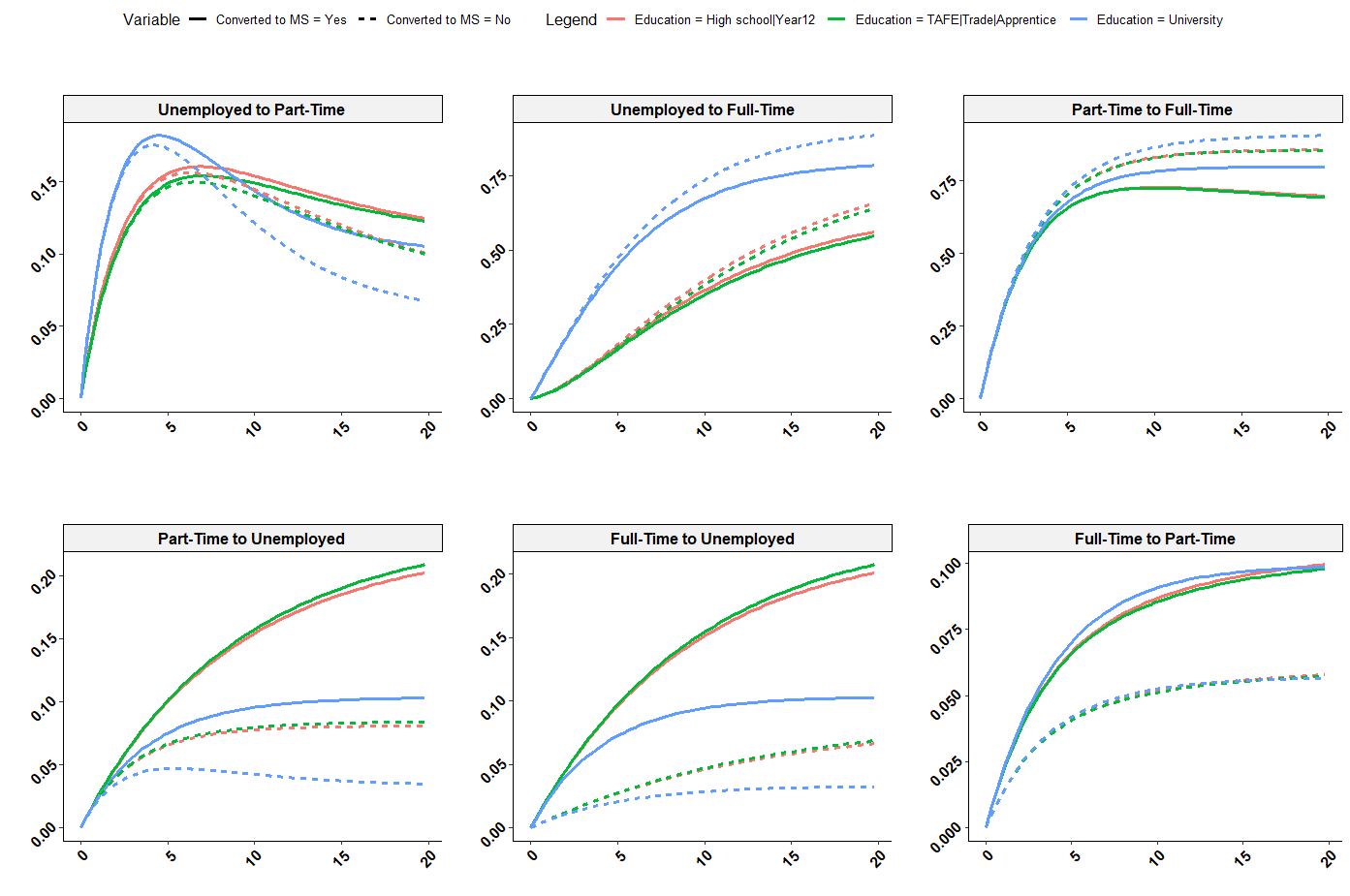


Supplementary Figure 1**.** Predicted employment transitions over a 20-year period for a participant who is 38 years old at the time of FCD based on the fitted final multivariable model by education level and progression to CDMS.


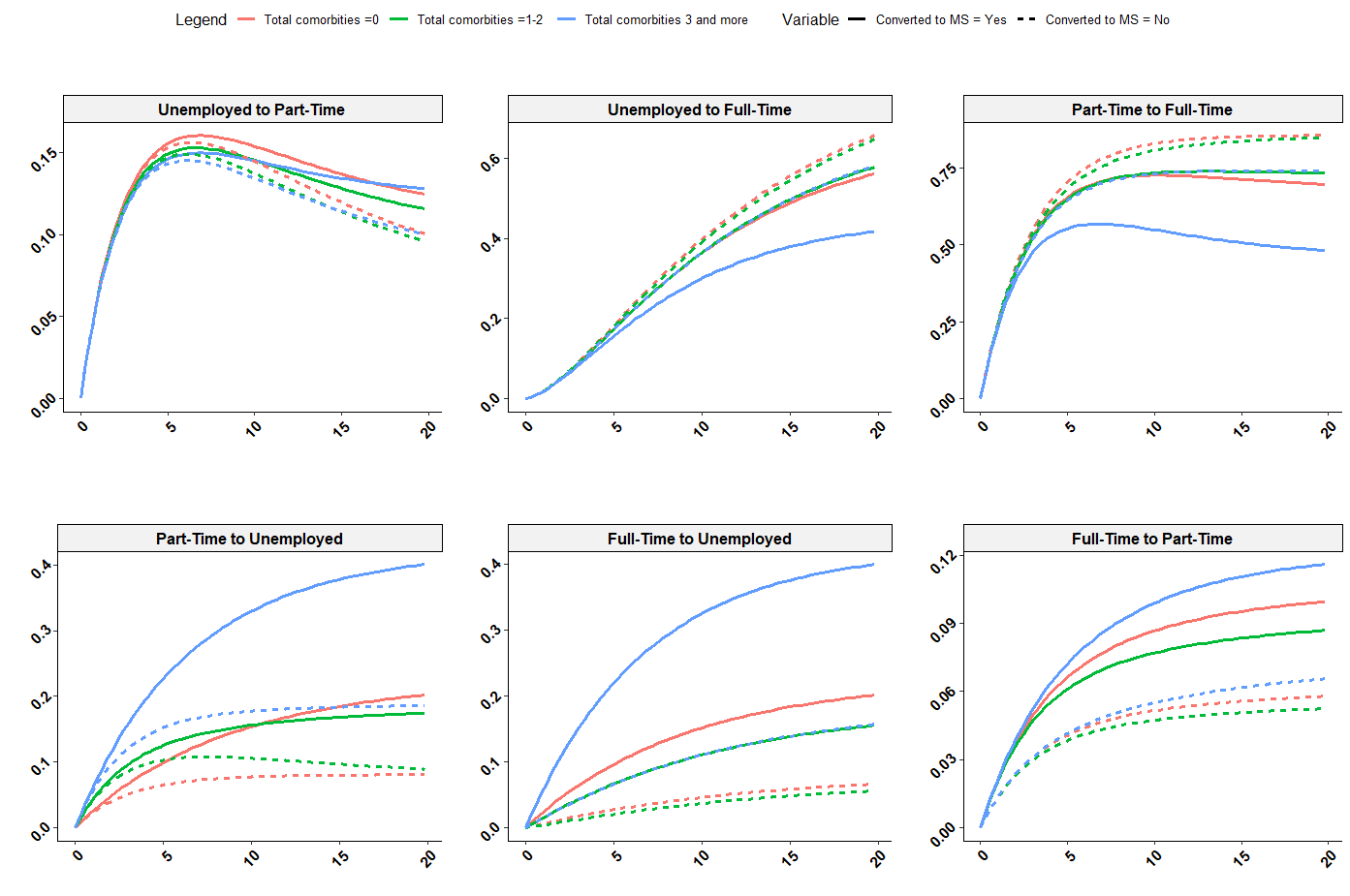


Supplementary Figure 2**.** Predicted employment transitions over a 20-year period for a participant who is 38 years old at the time of FCD based on the fitted final multivariable model by number of total comorbidities at baseline and progression to CDMS.
